# Sex Differences in Demographic and Pharmacological Factors in Alzheimer Patients With Dementia and Cognitive Impairments

**DOI:** 10.3389/fnbeh.2022.828782

**Published:** 2022-04-01

**Authors:** Oreoluwa O. Coker-Ayo, Samuel I. Nathaniel, Nicolas Poupore, Melissa J. Bailey-Taylor, Laurie Theriot Roley, Richard L. Goodwin, Brooks McPhail, Rebecca Russ-Sellers, Thomas I. Nathaniel

**Affiliations:** ^1^Department of Biology, University of South Carolina, Columbia, SC, United States; ^2^Department of Biology, North Greenville University, Tigerville, SC, United States; ^3^School of Medicine Greenville, University of South Carolina, Greenville, SC, United States; ^4^Prisma Health Upstate, Greer, SC, United States

**Keywords:** gender -, demography, Alzheimer’s Disease, dementia, cognitive impairment

## Abstract

**Objective:**

The current study investigates sex differences associated with pharmacological and demographic characteristics in Alzheimer patients (AD) with dementia (ADD) or mild cognitive impairment (MCI).

**Method:**

A retrospective analytical approach was used to analyze data from 45,696 AD patients with MCI or ADD. The univariate analysis was used to determine differences in demographic, and pharmacological characteristics for male and female ADD and MCI-AD patients. Multivariate analysis was used to predict specific pharmacological and demographic factors that are associated with male and female MCI and ADD patients.

**Result:**

In the adjusted analysis for male patients, Hispanics [0.166,0.020 – 1.355, *P* = 0.094] or African Americans [OR = 2.380, 95% CI,2.120 – 2.674, *P* < 0.001], were more likely to have MCI-AD and be treated with galantamine [OR = 0.559, 95% CI, 0.382 – 0.818, *P* = 0.003], donepezil [OR = 1.639, 95% CI,1.503 – 1.787, *P* < 0.001], rivastigmine [OR = 1.394, 95% CI,1.184 – 1.642, *P* < 0.001], olanzapine [OR = 2.727, 95% CI,2.315 – 3.212, *P* < 0.001], risperidone [OR = 2.973, 95% CI,2.506 – 3.526, *P* < 0.001], present with increasing age [1.075,1.071 – 1.079, *P* < 0.001], and are on tobacco use [OR = 1.150, 95% CI,1.054 – 1.254, *P* = 0.002]. For female patients, buspirone [OR = 0.767, 95% CI, 0.683 – 0.861, *P* < 0.001] and a history of alcohol (ETOH) use [OR = 0.484, 95% CI, 0.442 – 0.529, *P* < 0.001] were associated with MCI-AD. Increasing age [OR = 1.096, 95% CI, 1.093 – 1.100, *P* < 0.001], donepezil [OR = 2.185, 95% CI, 2.035 – 2.346, *P* < 0.001], memantine [OR = 2.283, 95% CI, 2.104 – 2.477, *P* < 0.001] aripiprazole [OR = 1.807, 95% CI, 1.544 – 2.113, *P* < 0.001] olanzapine [OR = 2.289, 95% CI, 1.986 – 2.640, *P* < 0.001] risperidone [OR = 2.548, 95% CI, 2.246 – 2.889, *P* < 0.001] buspirone [OR = 0.767, 95% CI, 0.683 – 0.861, *P* < 0.001] escitalopram [OR = 1.213, 95% CI,1.119 – 1.315, *P* < 0.001] African Americans [OR = 1.395, 95% CI, 1.268 – 1.535, *P* < 0.001] and tobacco use [OR = 1.150, 95% CI, 1.073 – 1.233, *P* < 0.001] were associated with ADD.

**Conclusion:**

Our findings reveal that MCI-AD patients were more likely to be Hispanics or African American males treated with rivastigmine, olanzapine and citalopram. African American females were associated with ADD and more likely to be treated with buspirone and presented with a history of ETOH. This finding suggests the need for a pharmacological treatment approach encompassing sex-sensitive strategies for MCI-AD and ADD patients.

## Introduction

In the elderly, AD is a commonly observed etiology of mild cognitive impairment (MCI) and early dementia (Lu et al., 2021), and both are characterized by cognitive impairment (Petersen, 2016). The significant difference between MCI and dementia is that in dementia, more than one cognitive domain is affected, resulting in interference in activities of daily living (Knopman and Petersen, 2014). The prognosis for MCI and dementia is an essential motivation for early accurate diagnosis, as in both, there is a risk for further cognitive decline. In persons over age 70 years, more than 13% are reported to present significant cognitive impairment to warrant a diagnosis of dementia (Knopman and Petersen, 2014). Although a diagnosis of MCI may be made and later rescinded because of improvement in cognition, once diagnosed with MCI, individuals are at greater risk for future decline than those who never had MCI (Knopman and Petersen, 2014; Langa and Levine, 2014). In contrast, persons with dementia almost invariably worsen over time (Petersen et al., 2010). In MCI associated with AD (MCI-AD) and dementia associated with AD (ADD), cognitive function is characterized by the Diagnostic and Statistical Manual of Mental Disorders (DSMMD) into 5 domains: (1) learning and memory, (2) language, (3) visuo-spatial, (4) executive, and (5) psychomotor (Tarawneh and Holtzman, 2012; Knopman and Petersen, 2014). For a diagnosis of MCI, only one of these domains must be impaired in order to make a diagnosis, whereas more than one domain are impaired to make a diagnosis of dementia (Tarawneh and Holtzman, 2012).

Females with MCI are reported to present with greater longitudinal rates of cognitive and functional decline than males (Tarawneh and Holtzman, 2012), and females make up almost two-thirds of AD patients in the United States (Hebert et al., 2013). The explanation provided for the higher cases of AD and rates of cognitive impairments in females is often linked to their greater longevity, and sociocultural factors (Mielke et al., 2014). The existing literature is far from conclusive and consists mainly of hypotheses that involve sex-specific biological and sex-specific sociocultural factors that increase females’ vulnerability over males (Knopman and Petersen, 2014). However, a deeper analysis of the extant literature indicates a more complex mechanism. In general, understanding sex-specific trends in ADD and MCI-AD points to preclinical, demographic and pharmacological factors that could reveal onset and differential outcomes between males and females (Knopman and Petersen, 2014; Dubois et al., 2016). Pharmacological treatment of MCI-AD is limited (Petersen et al., 1999). Clinical trials involving a wide range of substances for MCI have failed to show efficacy on primary and secondary outcome parameters for treatments (Karakaya et al., 2013), suggesting that most treatments are targeted at AD. Several trials of cholinesterase inhibitors (ChEIs) have been conducted in individuals with amnestic type MCI, which is likely due to underlying AD (Tricco et al., 2013; Matsunaga et al., 2019). Cholinesterase inhibitors – donepezil, rivastigmine and galantamine are approved medications for the treatment of dementia due to AD (Dou et al., 2018), and outcomes have so far produced modest benefits (Li et al., 2019). Therefore, the decision to treat ADD patients with a ChEI is based on the likelihood that AD was the underlying etiology (Grossberg et al., 2019), indicating that other medications combined with a ChEI for the treatment of symptoms of other than those found in MCI-AD and ADD patients.

Preclinical data suggest that second-generation antipsychotics (SGAs) could reduce cognitive impairments (Goldberg et al., 2007; Hill et al., 2010). Whether there is evidence of sex differences in the use of SGAs as a pharmacological treatment option for MCI-AD and AD is not fully understood. Knowledge about the efficacy and limitations of the antidementive drugs and ChEIs in different stages of AD indicates a critical role for the serotonergic system in memory retention and learning by interacting with the cholinergic dopaminergic, γ-aminobutyric acid (GABA)ergic and glutaminergic systems (Seyedabadi et al., 2014). Selective serotonin reuptake inhibitors (SSRIs) are approved in the treatment of depressive disorders (Strawn et al., 2018), and fluoxetine an SSRI, is reported to enhance cognitive performance in AD (Xie et al., 2019). SSRIs selectively target the solute carrier family 6 member 4 responsible for terminating the action of serotonin in the synaptic cleft, consequently increasing neurotransmitter availability in the synapse (Mdawar et al., 2020). While SSRIs have emerged as promising therapies to delay the onset of cognitive deterioration in AD patients, it is not clear whether there are sex-specific differences in treating ADD and MCI-AD patients with SSRIs.

Since our sample was restricted to ADD and MCI-AD patients, we assumed that more females than males might be affected, which is typical for the AD population (Viña and Lloret, 2010). Therefore, we hypothesized that males and females with ADD and MCI-AD differ regarding treatment with ChEIs or other medications including SSRIs and SGAs. The present study examined differences in patient demographics and pharmacological therapies in individuals treated with ChEIs, SSRIs and SGAs, and how this might contribute to sex differences between males and females with ADD and MCI-AD. Therefore, we determined sex-specific differences in ADD and MCI-AD patients undergoing ChEI, SSRIs and SGAs therapies. Moreover, since males and females present with differences in cognitive progression with females declining at much higher rates than males (Lin et al., 2015; Laws et al., 2016), we also determined specific demographic factors contributing to sex differences in patients who received ChEI, SSRIs, and SGAs.

## Materials and Methods

### Study Population

Retrospective data of patients diagnosed with MCI-AD and ADD patients (early dementia associated AD) were retrieved from Alzheimer’s database registry of the Prisma Health-Upstate (formerly known as Greenville Health System) from February 2016 to August 2021. The inclusion criteria were data from outpatients aged ≥ 40 years who met the requirements for the clinical diagnosis of MCI or early dementia, as defined in the DSMMD[26], and for possible AD according to the criteria of the National Institute of Neurological and Communicative Disorders and Stroke and the Alzheimer’s Disease and Related Disorders Association (Cacabelos, 2007). Data for MCI-AD patients assessed using Mini-Mental State Exam (MMSE), with scores indicating mild cognitive impairment, were included in this study, while those with scores indicating more severe impairments were excluded. Data for patients not fulfilling the diagnostic criteria for AD and early dementia were also excluded. Pharmacological, social, and demographic risk factors were collected from a single database. Data for patients taking ChEIs including donepezil, galantamine, and rivastigmine, were also collected from this source. In addition, we obtained data for patients taking SSRIs including citalopram, escitalopram paroxetine, memantine, trazodone, buspirone, valproate, SGA medications including antipsychotics such as aripiprazole, olanzapine, and risperidone. Other variables included in this study were tobacco use and alcohol (ETOH) use. ETOH use was determined based off any past consumption of ETOH regardless of time and amount of consumption. Tobacco use was recorded in a similar fashion. Demographic factors included the age, race, and ethnicity of subjects.

### Statistical Analysis

Univariate statistical analysis was used to determine demographic and pharmacological characteristics of patients with MCI-AD and ADD by sex. Discrete variables comparing MCI-AD and ADD patients were analyzed using the Man Whitney U or Pearson Chi-square test. Standard deviation, mean, and range were all calculated for continuous variables. The number and percentage of patients in that category were calculated for all discrete variables. The regression models were built using the established predictors from our univariate analysis using the backward selection method. This method was chosen because it allowed all the initially selected demographic and pharmacological risk factors to be included in the model and then systematically removed if they did not contribute to the overall significance of the model. The multicollinearity was determined for the interactive effects of variables using variance inflation factors (VIFs), with values > 5 has been reported to be suggestive of multicollinearity (Becker et al., 2015). Further, the validity of our model was tested using a Hosmer-Lemeshow test. The overall correct classification percentage and the area under the receiver operating curve (AUROC) for score prediction were determined to test the model’s sensitivity, specificity, and accuracy.

For each regression model, the dependent variables were MCI-AD or ADD, while the independent variables were the pharmacologic and demographic factors in patients with MCI-AD or ADD, stratified by sex. The regression models were developed separately for MCI-AD and ADD outcomes for males and females. Odd ratios at 0.5 significance level and 95% confidence interval (95% CI) were considered. The likelihood of being associated with MCI-AD or ADD was determined separately for male and female patients. The overall correct classification percentage and area under the Receiver Operating Curve (ROC) were used to determine the logistic regression model’s sensitivity, specificity, and accuracy for male and female patients with MCI-AD or ADD. Statistical analyses were performed using SPSS software ver. 26.0 (IBM, Armonk, NY, United States).

## Results

A total of 45,696 AD Patients with 18,153 males and 27,543 females were included in this study. As shown in Table 1, females were more likely to present with increasing age (69.72 ± 20.70 vs. 67.19 ± 22.00), less likely to use tobacco (41.9 vs. 65.6%), and alcohol (25.2 vs. 35.6) than males. Additionally, females were less likely to be taking ChEIs (27.8 vs. 29.9%) including donepezil (25.1 vs. 27.2%) and galantamine (0.5 vs. 0.9%). However, females were more likely to be taking SGAs (15.7 vs. 13.5%), including aripiprazole (5.7 vs. 4.7%) and risperidone (6.9 vs. 5.4%), but less likely to take olanzapine (5.5 vs. 6.0%). They were also more likely to be treated with SSRIs (33.6 vs. 28.3%) than males, specifically, citalopram (11.3 vs. 10.1%) and escitalopram (21.9 vs. 17.7%), memantine (17.3 vs. 15.9), and buspirone (11.2 vs. 7.0%).

**TABLE 1:** Sex differences of demographic and clinical characteristics in mild cognitive impairment and dementia patients.

Characteristic	Male	Female	

Number of patients	18153	27543	*P*-value
**Age Group: No. (%)**			
< 50	3283 (18.1)	4499 (16.3)	<0.001*^a^
50-59	1467 (8.1)	2687 (9.8)	
60-69	2777 (15.3)	3683 (13.4)	
70-79	4451 (24.5)	6066 (22.0)	
> = 80	6175 (34.0)	10608 (38.5)	
Mean ± SD	67.19 ± 22.00	69.72 ± 20.70	< 0.001*^b^
**Race: No (%)**			
White	14722 (81.1)	22300 (81.0)	< 0.001*^a^
Black	2465 (13.6)	4059 (14.7)	
Other	966 (5.3)	1184 (4.3)	
Hispanic Ethnicity: No. (%)	418 (2.3)	594 (2.2)	0.298
Tobacco	11402 (65.6)	11316 (41.9)	< 0.001*^a^
ETOH	6156 (35.6)	6784 (25.2)	< 0.001*^a^
Length of Stay	2.18 ± 7.63	1.70 ± 4.78	< 0.001*^b^
**Medications**			
Central acetylcholinesterase inhibitor	5425 (29.9)	7659 (27.8)	< 0.001*^a^
Donepezil	4943 (27.2)	6914 (25.1)	< 0.001*^a^
Galantamine	158 (0.9)	128 (0.5)	< 0.001*^a^
Rivastigmine	797 (4.4)	1197 (4.3)	0.820
Second Generation Antipsychotic	2447 (13.5)	4330 (15.7)	< 0.001*^a^
Aripiprazole	859 (4.7)	1582 (5.7)	< 0.001*^a^
Olanzapine	1091 (6.0)	1504 (5.5)	0.013*^a^
Risperidone	983 (5.4)	1894 (6.9)	< 0.001*^a^
Selective Serotonin Receptor Inhibitor	5140 (28.3)	9250 (33.6)	< 0.001*^a^
Citalopram	1835 (10.1)	3107 (11.3)	< 0.001*^a^
Escitalopram	3217 (17.7)	6019 (21.9)	< 0.001*^a^
Paroxetine	0 (0.0)	0 (0.0)	
Memantine	2880 (15.9)	4776 open (17.3)	< 0.001*^a^
Trazodone	0 (0.0)	0 (0.0)	
Buspirone	1272 (7.0)	3095 (11.2)	< 0.001*^a^
Valproate	0 (0.0)	0 (0.0)	

Results for continuous variables are presented as Mean ± SD, while discrete data are presented as percentage frequency. Pearson’s Chi-Square is used to compare sex differences between demographic and clinical characteristics in patients with mild cognitive impairment and dementia. ^a^Pearson’s Chi-Squared test; ^b^Student’s T test; *P-value < 0.05.

A total of 19,495 females presented with MCI-AD while 8,048 females presented ADD, whereas 13,569 males presented with MCI while 8048 males presented with ADD (Table 2). Females with ADD were more likely to be younger (84.35 ± 9.69 vs. 63.68 ± 21.02), Caucasian (82.1 vs. 80.5%), and less likely to be Hispanic (1.3 vs. 2.5%). Additionally, females with ADD presented with lower rates of tobacco (37.2 vs. 43.9%) and ETOH (12.8 vs. 38.4%) use. Higher lengths of stay (2.16 ± 4.22 vs. 1.51 ± 4.98) were found in females with dementia compared to MCI. They were also more likely to take ChEIs (51.4 vs. 18.1%), specifically donepezil (45.6 vs. 18.7%), galantamine (0.8 vs. 0.3%), and rivastigmine (9.3 vs. 2.3%). In addition, females with ADD were more likely to take SGAs (20.5 vs. 13.8%) including aripiprazole (4.8 vs. 6.1%), olanzapine (7.9 vs. 4.5%), risperidone (10.8 vs. 5.3%), and SSRIs (35.4 vs. 32.8%) such as citalopram (12.6 vs. 10.8%), escitalopram (23.9 vs. 21.0%), and memantine (34.8 vs. 10.1%) with the exception of buspirone (8.2 vs. 12.5%).

**TABLE 2:** Demographic and clinical characteristics of mild cognitive impairment versus dementia in patients stratified by sex.

	Male			Female		
			
Characteristic	Mild Cognitive Impairment	Dementia		Mild Cognitive Impairment	Dementia	

Number of patients	13569	4584	*P*-value	19495	8048	*P*-Value
**Age Group: No. (%)**						
< 50	3230 (23.8)	53 (1.2)	<0.001*^a^	4463 (22.9)	36 (0.4)	<0.001*^a^
50-59	1375 (10.1)	92 (2.0)		2644 (13.6)	43 (0.5)	
60-69	2351 (17.3)	426 (9.3)		3227 (16.6)	456 (5.7)	
70-79	3301 (24.3)	1150 (25.1)		4421 (22.7)	1645 (20.4)	
> = 80	3312 (24.4)	2863 (62.5)		4740 (24.3)	5868 (72.9)	
Mean ± SD	62.49 ± 22.82	81.08 ± 10.86	< 0.001*^b^	63.68 ± 21.02	84.35 ± 9.69	< 0.001*^b^
**Race: No (%)**						
White	10993 (81.0)	3729 (81.3)	< 0.001*^a^	15696 (80.5)	6604 (82.1)	< 0.001*^a^
Black	1743 (12.8)	722 (15.8)		2887 (14.8)	1172 (14.6)	
Other	833 (6.1)	133 (2.9)		912 (4.7)	272 (3.4)	
Hispanic Ethnicity: No. (%)	366 (2.7)	52 (1.1)	< 0.001*^a^	491(2.5)	103 (1.3)	< 0.001*^a^
Tobacco	8281 (64.2)	3121 (69.5)	< 0.001*^a^	8389 (43.9)	2927 (37.2)	< 0.001*^a^
ETOH	5003 (39.1)	1153 (25.7)	< 0.001*^a^	5775 (30.4)	1009 (12.8)	< 0.001*^a^
Length of Stay	2.09 ± 7.79	2.45 ± 7.13	0.006*^b^	1.51 ± 4.98	2.16 ± 4.22	< 0.001*^b^
**Medications**						
Central acetylcholinesterase inhibitor	3032 (22.3)	2393 (52.2)	< 0.001*^a^	3522 (18.1)	4137 (51.4)	< 0.001*^a^
Donepezil	2807 (20.7)	2136 (46.6)	< 0.001*^a^	3246 (16.7)	3668 (45.6)	< 0.001*^a^
Galantamine	100 (0.7)	58 (1.3)	< 0.001*^a^	67 (0.3)	61 (0.8)	< 0.001*^a^
Rivastigmine	388 (2.9)	409 (8.9)	< 0.001*^a^	447(2.3)	750 (9.3)	< 0.001*^a^
Second Generation Antipsychotic	1598 (11.8)	849 (18.5)	< 0.001*^a^	2682 (13.8)	1648 (20.5)	< 0.001*^a^
Aripiprazole	698 (5.1)	161 (3.5)	< 0.001*^a^	1198 (6.1)	384 (4.8)	< 0.001*^a^
Olanzapine	672 (5.0)	419 (9.1)	< 0.001*^a^	871(4.5)	633 (7.9)	< 0.001*^a^
Risperidone	577 (4.3)	406 (8.9)	< 0.001*^a^	1026 (5.3)	868 (10.8)	< 0.001*^a^
Selective Serotonin Receptor Inhibitor	3753 (27.7)	1387 (30.3)	< 0.001*^a^	6397 (32.8)	2853 (35.4)	< 0.001*^a^
Citalopram	1301 (9.6)	534 (11.6)	< 0.001*^a^	2096 (10.8)	1011 (12.6)	< 0.001*^a^
Escitalopram	2333 (17.2)	884 (19.3)	0.001*^a^	4097 (21.0)	1922 (23.9)	< 0.001*^a^
Paroxetine	0 (0.0)	0 (0.0)		0 (0.0)	0 (0.0)	
Memantine	1365 (10.1)	1515 (33.0)	< 0.001*^a^	1973 (10.1)	2803 (34.8)	< 0.001*^a^
Trazodone	0 (0.0)	0 (0.0)		0 (0.0)	0 (0.0)	
Buspirone	996 (7.3)	276 (6.0)	0.002*^a^	2436 (12.5)	650 (8.2)	< 0.001*^a^
Valproate	0 (0.0)	0 (0.0)		0 (0.0)	0 (0.0)	

Results for continuous variables are presented as Mean ± SD, while discrete data are presented as percentage frequency. Pearson’s Chi-Square is used to compare differences between demographic and clinical characteristics in groups either mild cognitive Impairment or dementia stratified by sex. ^a^Pearson’s Chi-Squared test; ^b^Student’s T test; *P-value < 0.05.

Males with ADD were more likely to be older (81.08 ± 10.86% vs. 62.49 ± 22.82%) and Caucasian (81.3 vs. 81.0%), and less likely to be Hispanic (1.1 vs. 2.7%), with higher rates of tobacco use (69.5 vs. 64.2%) and lower rates of ETOH use (25.7 vs. 39.1%). Males with ADD were also more likely to take ChEIs (52.2 vs. 22.3%), specifically donepezil (46.6 vs. 20.7%), galantamine (1.3 vs. 0.7%), and rivastigmine (8.9 VS. 2.9%). They were more likely to take SGAs (18.5 vs.11.8%) including olanzapine (9.1 vs. 5.0%), risperidone (8.9 vs. 4.3%), and SSRIs (30.3 vs. 27.7%) including citalopram (11.6 vs. 9.6%), escitalopram (19.3 vs. 17.2%), memantine (33.0 vs. 10.1% and buspirone (6.0 vs. 7.3%) with the exception of (aripiprazole (3.5 vs. 5.1%).

**FIGURE 1:**
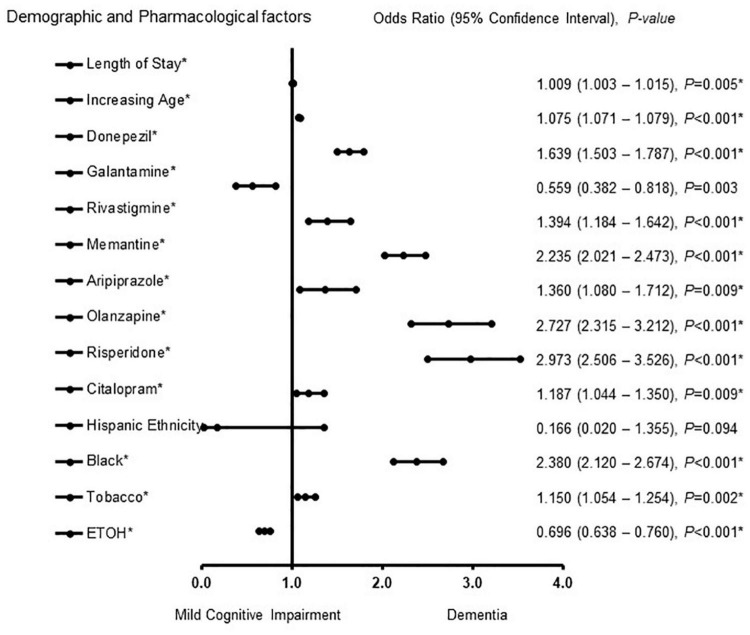
Demographic and pharmacological factors associated with mild cognitive impairment and Alzheimer patients with dementia in males. Adjusted OR < 1 denote factors that are associated with mild cognitive impairment while OR > 1 denote factors that are associated with dementia. Hosmer-Lemeshow test (*P* < 0.001*), Cox & Snell (*R^2^* = 0.217). The overall classified percentage of 76.6% was applied to check for fitness of the logistic regression model. *Indicates statistical significance (*P* < 0.05) with a 95% confidence interval.

Figure 1 shows demographic and pharmacological factors associated with MCI-AD and ADD in male patients. Galantamine [OR = 0.559, 95% CI, 0.382 – 0.818, *P* = 0.003], and Hispanic ethnicity [OR = 0.166, 95% CI, 0.020 – 1.355, *P* = 0.094] were associated with MCI. Length of stay [OR = 1.009, 95% CI, 1.003 – 1.015, *P* = 0.005], increasing age [OR = 1.075, 95% CI, 1.071 – 1.079, *P* < 0.001], donepezil [OR = 1.639, 95% CI, 1.503 – 1.787, *P* < 0.001], rivastigmine [OR = 1.394, 95% CI, 1.184 – 1.642, *P* < 0.001], memantine [OR = 2.235, 95% CI, 2.021 – 2.473, *P* < 0.001], aripiprazole [OR = 1.360, 95% CI, 1.080 – 1.712, *P* = 0.009], olanzapine [OR = 2.727, 95% CI, 2.315 – 3.212, *P* < 0.001], risperidone [OR = 2.973, 95% CI, 2.506 – 3.526, *P* < 0.001], citalopram [OR = 1.187, 95% CI, 1.044 – 1.350, *P* = 0.009] African American racial group [OR = 2.380, 95% CI, 2.120 – 2.674, *P* < 0.001], and a history of tobacco use [OR = 1.150, 95% CI, 1.054 – 1.254, *P* = 0.002] were associated with dementia. The predictive power of the regression model was moderately strong. The area under the curve (AUORC) is 0.805, 95% CI, *P* < 0.001.

**FIGURE 2:**
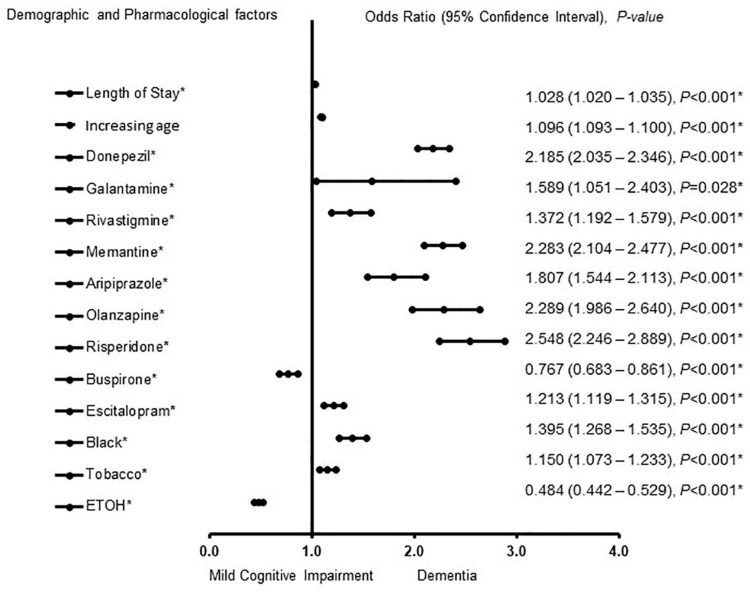
Demographic and pharmacological factors that were associated with mild cognitive impairment and Alzheimer patients with dementia in females. Adjusted OR < 1 denote factors that are associated with mild cognitive impairment while OR > 1 denote factors that are associated with dementia. Hosmer-Lemeshow test (*P* < 0.001*), Cox & Snell (*R^2^* = 0.307). The overall classified percentage of 70.7% was applied to check for fitness of the logistic regression model. *Indicates statistical significance (*P* < 0.05) with a 95% confidence interval.

The factors associated with MCI-AD and ADD in females were also determined (Figure 2). Buspirone [OR = 0.767, 95% CI, 0.683 – 0.861, *P* < 0.001] and a history of ETOH use [OR = 0.484, 95% CI, 0.442 – 0.529, *P* < 0.001] were associated with MCI. Length of stay [OR = 1.028, 95% CI, 1.020 – 1.035, *P* < 0.001], increasing age [OR = 1.096, 95% CI, 1.093 – 1.100, *P* < 0.001], donepezil [OR = 2.185, 95% CI, 2.035 – 2.346, *P* < 0.001], galantamine [OR = 1.589, 95% CI, 1.051 – 2.403, *P* = 0.028] rivastigmine [OR = 1.372, 95% CI, 1.192 – 1.579, *P* < 0.001] memantine [OR = 2.283, 95% CI, 2.104 – 2.477, *P* < 0.001] aripiprazole [OR = 1.807, 95% CI, 1.544 – 2.113, *P* < 0.001] olanzapine [OR = 2.289, 95% CI, 1.986 – 2.640, *P* < 0.001] risperidone [OR = 2.548, 95% CI, 2.246 – 2.889, *P* < 0.001], escitalopram [OR = 1.213, 95% CI, 1.119 – 1.315, *P* < 0.001] African American racial group [OR = 1.395,1.268 – 1.535, *P* < 0.001] and a history of tobacco use [OR = 1.150, 95% CI, 1.073 – 1.233, *P* < 0.001] were associated with ADD. The strength of the model was found to be moderately strong. The area under the curve (AUORC) is 0.853, 95% CI, P < 0.001.

## Discussion

ADD and MCI-AD patients represent a significant clinical group as they are at increased risk of worsening cognitive functions and are an ideal target for therapeutic interventions. Since biological changes typical of AD have been found in MCI patients (Frisoni et al., 2010), and pharmacological treatment of MCI due to AD is limited, the decision to treat patients with a ChEI would depend on whether an underlying etiology of AD could be assessed. The current study evaluated sex differences in ADD and MCI-AD patients treated with ChEI, SSA, and SGA therapies. In the univariate analysis, our findings revealed that more female patients presented with ADD and MCI-AD when compared with males. Moreover, MCI-AD females were more likely to be taking SGAs, including aripiprazole and risperidone but less likely to take olanzapine. In addition, females were more likely to be treated with SSRIs, specifically citalopram escitalopram, memantine, and buspirone.

In the adjusted analysis for males, Hispanic males taking ETOH treated with galantamine were associated with MCI-AD. In contrast, African American males with an increasing length of stay for treatment, increasing age treated with donepezil, rivastigmine, memantine, aripiprazole, olanzapine, risperidone and citalopram were associated with ADD. For females, buspirone and a history of ETOH use were associated with MCI-AD, while African American females, and an increased length of stay, increasing age, and treatment with donepezil, galantamine, rivastigmine, memantine, aripiprazole, risperidone, buspirone and escitalopram were associated with ADD.

Pharmacologic treatments for AD with donepezil, galantamine, and rivastigmine include targeting the primary manifestations that have cognitive impairments observed in both ADD and MCI-ADD patients (Cacabelos, 2007). In general, ChEIs reduce acetylcholine breakdown in the brain and are considered a treatment option for AD. They also offer a feasible therapeutic target to stabilize cognitive functions (Stanciu et al., 2020). Donepezil, is a ChEI known to improve cerebral blood flow (CBF) to enhance memory (Kogure et al., 2017). Rivastigmine is a brain-selective inhibitor of “pseudo-irreversible” AChE, and its metabolism is independent of the cytochrome P450 system (Li et al., 2019). Galantamine is a newly available cholinergic drug that counteracts AD by specifically and reversibly inhibiting acetylcholinesterase (AChE) and altering the nicotinic cholinergic receptors, thereby reducing central cholinergic neurotransmission (Li et al., 2019).

Treatment outcomes of ChEIs are reported to be controversial. For example, donepezil, galantamine, and rivastigmine are reported to stabilize or slow the decline in cognition and improve cognition for donepezil- compared with galantamine-treated patients (Jones et al., 2004). Adjusted indirect comparisons suggest that donepezil and rivastigmine may be slightly more efficacious than galantamine (Hansen et al., 2008). Other studies indicate that galantamine has potent therapeutic effects on all aspects of AD, but donepezil and rivastigmine do not have effective therapeutic effects on some aspects of cognitive function (Li et al., 2019). We observed that males with MCI-AD were only treated with galantamine. In contrast, females with MCI-AD did not receive any ChEIs, indicating differences in the use of ChEIs as a treatment option for males and females with MCI-AD.

While our current data cannot explain the reason that females with MCI-AD were not treated with any ChEIs therapies in our data set, ChEIs are reported to slightly delay the loss of brain function in people who have mild to moderate AD; however, they do have side effects such as nausea, dizziness, vomiting, diarrhea, dizziness, asthenia and anorexia, all symptoms linked to cholinergic overstimulation (Imbimbo, 2001). Therefore, the adverse events may outweigh the benefits, such that ChEIs produce a small gift on several cognitive function scales (Hitzeman, 2006; Bailey-Taylor et al., 2022) in female patients. Moreover, our finding that males and females with ADD were more likely to receive donepezil, galantamine, and rivastigmine reveals a robust comparable approach in using ChEIs for ADD males and females. Since females present with higher rates of clinically diagnosed cases of dementia and AD (Beam and Kim, 2020), more excellent longitudinal rates of cognitive and functional decline in MCI than males (Tarawneh and Holtzman, 2012), a comparable robust approach in ChEIs may offer a robust approach to manage and reduce the high rates of ADD in females.

MCI-AD and ADD are not a part of the normal aging process (Lo, 2017). In individuals with ADD, an impairment in cognitive function that results in mental decline that is sufficiently severe to disrupt their activities of daily life (Popp et al., 2015). The cognitive efficacy of antipsychotics has gained more research attention in recent years, as aripiprazole (Kohen et al., 2010), risperidone, or olanzapine (Vigen et al., 2011) improved cognitive functions in ADD patients. Loss of cognitive functions in dementia patients is characterized by a loss of more than one cognitive domain including learning and memory, language, visuo-spatial, executive and psychomotor (Tarawneh and Holtzman, 2012). In contrast, for MCI, only one of these domains must be impaired to make a diagnosis (Tarawneh and Holtzman, 2012). Our finding that SGAs including aripiprazole, olanzapine, risperidone were administered to both males and females with ADD is supported by previous studies (Seeman, 2004; Fulone et al., 2021), which found that antipsychotic use should not differentiate between male and female ADD patients. However, human studies (Beierle et al., 1999; Pérez et al., 2003) have shown that the pharmacokinetics and the pharmacodynamics of drugs differ in females and males and are influenced by sex-specific factors such as body habitus, diet, concurrent medications, and hormonal transitions. Furthermore, neurotransmitter levels diminish with age at different rates in females than in males (Peters, 2006). Some antipsychotic drug treatments have side effects, such as weight gain, which is more worrisome for females than males (Seeman, 2020). Therefore, while females may require same antipsychotic medication like males to achieve a better outcome, it may be at the expense of a higher side effect burden, precisely hormonal and metabolic side effects (Seeman, 2004). Therefore, sex-specific treatment regimens need to be developed to optimize outcomes in the use of SGAs for ADD patients.

We observed that males with ADD were more likely to be treated with memantine and citalopram, while females were more likely to be treated with memantine, buspirone, and escitalopram. While cholinergic dysfunction was long thought to be the sole contributor to AD symptomatology (Mdawar et al., 2020), growing evidence supports the contributory role of a dysfunctional monoaminergic system (Šimić et al., 2017). The serotonergic system plays a pivotal role in memory retention and learning by interacting with the cholinergic, dopaminergic, γ-aminobutyric acid (GABA)ergic and glutaminergic systems (Kandimalla and Reddy, 2017). Buspirone, escitalopram and citalopram are serotonin norepinephrine reuptake inhibitors (SNRIs) commonly used in elderly males and females, due to their tolerability and safety profile (Crocco et al., 2017). Memantine, a low-affinity non-competitive NMDA receptor antagonist, is the only glutamatergic drug approved for the treatment of moderate to severe cognitive symptoms of AD (Tariot et al., 2004). Memantine can be used in addition to acetylcholinesterase inhibitors in patients with AD (Scarpini et al., 2003). This specific combination is reported to delay the progression of dementia by preventing the pathological activation of NMDA receptors (Parsons et al., 2013). Therefore, our finding supports existing studies (Scarpini et al., 2003; Parsons et al., 2013) that memantine can be used for the initial therapy of cognitive functions in dementia patients.

Several studies support our finding that African American males and females with a history of ETOH use, increasing length of stay for treatment, and increasing age were associated with dementia (Langa et al., 2017). The higher rates of dementia among African-Americans contribute to an increased length of stay for care (Hill et al., 2015). There is also evidence that females have higher rates of dementia than males, mainly because females live longer (Mensah et al., 2005). Therefore, while disparities may be reduced by increasing levels of cognitive reserve and management of disease among blacks (Chen and Zissimopoulos, 2018), there also remains a complex combination of socioeconomic and cultural factors associated with these disparities. Health disparities often are seen through the lens of access to care or resources. However, a lack of diversity in clinical therapeutic development means that surmounting access barriers will not reduce disparities if therapeutics target only a small fraction of the diverse population. Future studies on factors associated with racial/ethnic differences in dementia risk should also focus on treatment options for racial and ethnic minorities by recruiting various participants into clinical trials of existing or new therapeutics.

## Limitations

Since this is a retrospective study, some potential limitations should be considered while interpreting the results of this study. The retrospective data were from a single institution; therefore, the results cannot be extrapolated to other institutions. In addition, data collection using electronic medical records could introduce human error as data from some study patients may have been excluded, which could have altered the results. Moreover, data on the systematic evaluation of behavioral disorders was not available since behavioral disorders, and the drugs used to treat them are usually considered for the prescription of ChEIs and other medications. Also, data on MMSE and CDR were not available to determine disease progression and behavioral alterations. In addition, information on the duration for the ChEIs, SSRSs, SGAs and data for apolipoprotein E (APOE) was not included in the database. Analyzing apolipoprotein E (APOE) in future studies will help determine sex differences or similarities and an increased risk at younger ages. It will also help to determine whether E (APOE) may contribute to cognitive change and differ across different demographic groups. All of our subgroup analyses were predetermined, and our analyses were repeated several times to eliminate the possibility of type 1 statistical errors. Finally, while this is a single study, the demonstration of consistent sex disparities in the demographic and pharmacological characteristics increases the generalizability of our findings.

## Conclusion

In our findings, Hispanic males treated with galantamine were associated with MCI, while African American males with the increasing length of stay, increasing age, and treated with donepezil, rivastigmine, memantine, aripiprazole, olanzapine, risperidone and citalopram were associated with dementia. For females, Buspirone and history of ETOH use were associated with MCI. In contrast, African American females, with an increased length of stay, increasing age, treatment with donepezil, galantamine, rivastigmine, memantine, aripiprazole, risperidone, buspirone, and escitalopram were associated with MCI-AD. Therefore, we observed differences and similarities in demographic factors and pharmacological therapies for males and females with MCI and dementia AD patients. These findings will hopefully facilitate developments in pharmacological treatment options of cognitive symptoms of dementia and MCI impairment due to AD in future studies. Furthermore, our results highlight the importance of taking sex into account in the clinical trials for ChEI, SGAs and SSRIs pharmacological agents for MCI and dementia patients with AD.

## Data Availability Statement

The original contributions presented in the study are included in the article/supplementary material, further inquiries can be directed to the corresponding author/s.

## Ethics Statement

This is a retrospective data collection. This study was approved by the Institutional Review Board of PRISMA Health institutional committee for ethics (approval number: 00052571). All data were fully anonymized before they were accessed. Patients’ data used in our retrospective analysis were from PRISMA Health Alzheimer data registry. Written informed consent for participation was not required for this study in accordance with the national legislation and the institutional requirements.

## Author Contributions

OC-A, SN, MB-T, NP, LR, and TN designed the concept, experiment and data analysis. At the same time, RG, BM and RR-S critically revised the drafts, interpreted the results, read and approved the last version of this manuscript. All authors have read and approved the manuscript.

## Conflict of Interest

The authors declare that the research was conducted in the absence of any commercial or financial relationships that could be construed as a potential conflict of interest.

## Publisher’s Note

All claims expressed in this article are solely those of the authors and do not necessarily represent those of their affiliated organizations, or those of the publisher, the editors and the reviewers. Any product that may be evaluated in this article, or claim that may be made by its manufacturer, is not guaranteed or endorsed by the publisher.

## Abbreviations

AD: Alzheimer’s disease
AChE: Acetylcholinesterase inhibitors
IRB: Institutional Review Board
APOE: Apolipoprotein E
CDR: clinical dementia rating
ChEI: Cholinesterase inhibitor
EOAD: Early-onset Alzheimer’s disease
LOAD: Late-onset Alzheimer’s disease
NSAIDs: non-steroidal anti-inflammatory drugs
NMDA: N-methyl-D-aspartate
ETOH: Ethanol
MMSE: mini mental exam
SSRIs: Selective Serotonin Reuptake Inhibitor
*AUROC*: Area Under the Receiver Operating Characteristics
ROC: receiver operating characteristic curve
OR: Odd ratio
SGA: second generation antipsychotics
VIF: Variance Inflation factor.
